# ‘I would have killed myself had it not been for this service’: qualitative experiences of NHS and third sector crisis care in the UK

**DOI:** 10.1192/bjo.2025.30

**Published:** 2025-05-14

**Authors:** Laura Sambrook, Anna Balmer, Jackie Tait, Peter Ashley-Mudie, Jason C. McIntyre, Amrith Shetty, Rajan Nathan, Pooja Saini

**Affiliations:** Faculty of Health, Liverpool John Moores University, Liverpool, UK; Cheshire and Wirral Partnership NHS Foundation Trust, Chester, UK

**Keywords:** Mental health, crisis, qualitative research

## Abstract

**Background:**

More people than ever are receiving support for mental health crises, and instances of suicide continue to grow. Mental health funding has recently increased, focusing on improving services that provide an alternative to emergency departments, such as urgent helplines and crisis cafés. However, there is a lack of literature examining the efficacy of these services, despite research suggesting they may be associated with lower hospital admission rates.

**Aims:**

We aimed to evaluate the perspectives of people with lived experience of accessing a variety of mental health crisis services in the UK.

**Method:**

One-to-one interviews were conducted with 25 individuals as part of a qualitative grounded theory analysis.

**Results:**

The following themes were identified as important for recovery: more than a diagnosis (a need for person-centred care); instilling hope for the future (access to creative spaces and community); and a safe space for recovery (out-of-hours crisis cafés). Many have credited crisis cafés with saving their lives and felt there should be increased funding provided for collaboration between the National Health Service (NHS) and the third sector. Participants highlighted the need for interim support for those awaiting therapy via the NHS and continuity of care as key areas for improvement.

**Conclusions:**

NHS services are struggling to meet the mental health needs of the population, resulting in lengthy waiting times for therapy and an over-reliance on the third sector. While crisis cafés are currently provided at a low cost and appear to result in satisfaction, policymakers must ensure they receive adequate funding and do not become overburdened.

One in four adults in England experiences at least one diagnosable mental health condition every year,^1^ with mental health problems representing the largest single cause of disability in the UK as a whole.^2^ More people than ever are receiving support for mental health crises and, tragically, the numbers of those ending their life by suicide have significantly increased over the past decade.^3^ In response, the National Health Service (NHS) in England^2^ has identified mental health as a key focus in recent years.^4^ Although commitments to improving NHS-funded services have been made, and funding for mental health services has increased, demand continues to outstrip investment and many targets are not being met.^4,5^ Historically, funding for mental health services has been insufficient relative to population needs, and services are now facing demands that were not anticipated at the time of the NHS Long-Term Plan,^6^ such as inflation and the impact of the COVID-19 pandemic.^5^ Government figures provide evidence that the number of people unable to access treatment in a timely manner continues to increase, but NHS services do not have the resources they need to respond to such growth in demand.^4^

## Current provision

In England, mental health support is provided by a mixture of NHS organisations, third sector (non-governmental, non-profit) enterprises, local authorities and independent providers. Services can be conceptualised as primary (mild to moderate), secondary (complex and concurrent) and tertiary (severe and enduring).^6^ Crisis services support individuals requiring urgent mental health support, including home treatment teams, crisis cafés, helplines and liaison mental health services in emergency departments^7^ (see Table 1). As well as pledging to invest £2.3 billion of additional funding into mental health services per year, an additional £57 million will be invested in suicide prevention, including new crisis cafés and improvements to crisis lines and emergency departments.^3^ The aim is for all crisis services to be ‘open access’ by 2028,^2^ meaning that individuals can self-refer, with a new government strategy aiming to reduce suicide rates, improve treatment for self-harm and enhance support for people bereaved by suicide.^7^

**Table 1:** Mental health crisis services and their definitions

Crisis service	Purpose and role of service
Home treatment team	To support people experiencing symptoms of such severity that, without the involvement of a home-based team, hospitalisation would be necessary
Crisis house	To offer safe short-term accommodation and support when home treatment is not suitable, or as a short-term alternative to hospital admission
Crisis café	To provide out-of-hours, community-based assessment and immediate support, intended to reduce pressure on emergency departments
Crisis helpline	To provide crisis response, triage and immediate emergency counselling via telephone only, usually by trained volunteers
Street triage team	A partnership scheme between police and approved mental health professionals to provide advice and support for people experiencing a mental health crisis
Liaison mental health services in emergency departments	To provide mental health assessments to individuals receiving care at emergency departments and who have been referred due to concerns surrounding their mental health and well-being
Acute day unit	A non-residential day hospital providing support, activity and therapeutic groups for people in crisis.

Crisis cafés are community-based services that provide an informal, non-clinical and accessible setting, aiming to reduce pressure on emergency departments.^8^ These typically operate outside of office hours and allow people to enter without an appointment.^8^ They are often led by staff who do not have professional mental health qualifications^9^, and include volunteers within their workforce.^10^ They are intended to support anyone in a self-defined crisis, irrespective of diagnosis, and are designed to be accessible at the first signs of crisis and before an individual becomes so unwell that they require in-patient treatment.^9^ Although there is a paucity of peer-reviewed literature examining the efficacy of crisis cafés, one recent study suggests that the addition of a crisis café as part of a mental health system may be associated with a 7.8% lower hospital admission rate.^11^ One study examined the attitudes of crisis café managers, finding that factors such as accessibility, person-centred care and quantity and quality of staff influenced the success of their services.^12^ However, literature focusing on patients’ experiences of these facilities is lacking.

## Complex mental health needs

While there is a wide array of services available for people with mental health problems, some have such complex mental health (CMH) needs that routinely available services are not best placed to address their needs.^13^ Research suggests that these individuals are not best served by community-based service delivery models,^13,14^ highlighting the need to examine their experiences of care. This cohort have often been in contact with mental health services for many years and have experienced acute psychiatric admissions and rehabilitation services.^15,16^ The majority have experienced psychosis, treatment resistance, severe negative symptoms and cognitive impairments.^17^ Compared with the general population, people with complex diagnoses such as schizophrenia have a reduced life expectancy of 10–25 years^18^ and higher premature mortality rates,^19^ with suicide a common cause of death.^20,21^ They are therefore more likely to engage with a wide variety of mental health services, including crisis support. People with CMH needs accessing the service have often been excluded from participating in service evaluation despite their ability to offer useful insight, and therefore it was deemed imperative to represent this group within our sample.

## Research aims

This study aimed to evaluate the perspectives of people with lived experience of accessing a variety of mental health services in the UK, with a focus on crisis care. The research addresses a gap in the literature because, to our knowledge, no qualitative studies have explored individuals’ experiences of third sector crisis support, far less offered a comparison between such services and other community-based and in-patient care. We aimed to provide insight into the experiences of people accessing mental health care in the UK, explore the strengths and limitations of third sector and NHS-based crisis support and provide recommendations for improving future practice.

## Method

### Design

One-to-one interviews were conducted to explore the experiences and perspectives of people engaging with mental health crisis services in the UK, as part of a qualitative grounded theory analysis.^22^ Interviews were semi-structured^23^ to allow for similar questions to be asked of all participants while allowing for flexibility if pertinent lines of inquiry arose.

### Study setting

The study took place at Cheshire and Wirral Partnership (CWP) NHS Foundation Trust. The Trust provides a range of community and in-patient physical and mental healthcare services, as well as providing care to a specific cohort of people with CMH needs. CMH is a broad term used to describe those who receive packages of care commissioned by NHS Cheshire integrated care systems in either an in-patient or community setting. This study included individuals who were detained under either Section 17 of the Mental Health Act or Section 117 Aftercare, or who had learning disabilities, acquired brain injuries or physical disabilities.

### Ethics statement

The authors assert that all procedures contributing to this work comply with the ethical standards of the relevant national and institutional committees on human experimentation, and with the Helsinki Declaration of 1975 as revised in 2013. All procedures involving human patients were approved by the NHS Health Research Authority and Research Ethics Committee: Integrated Research Application System prior to study commencement (REC Ref: 21/WM/0020 and 22/EM/0201).

### Participants

In total, 25 people were interviewed. It was a requirement of the study that participants were engaging with mental health services at the time of interview, either as in-patients or in the community. Participants were excluded if they were under the age of 18 years, unable to provide written informed consent or deemed lacking the capacity to consent by staff.

### Materials

Participants were provided with an information sheet and consent form to sign prior to taking part in the study. An interview schedule was developed with input from relevant stakeholders comprising CWP representatives, public and patient members, commissioners and the local authority (see Appendix 1). It was designed to facilitate discussions with participants about their experiences of contact with mental health services, as well as the care received. Prompts were included to guide the discussion if necessary, covering areas such as involvement in decision-making, psychological support and relationships with staff. Table 2 lists the topics and questions used within the interviews.

**Table 2:** Interview schedule questions

Overall topic	Questions
Emergency departments	Can you tell me about your experiences of attending hospital in a crisis?
Mental health services	Can you tell me about your experiences of mental health services more generally?
Community mental health services	Have you been cared for by the community mental health team? If yes, how was your experience?
In-patient mental health services	If you have been an in-patient, how did you find the decisions that were made in respect of your care?
Crisis line	Have you ever used the crisis line? If yes, how was your experience?
Crisis cafés	Have you ever utilised a crisis café? If yes, how was your experience?

### Procedures

Individuals were identified by CWP clinicians as being suitable to participate in the study, then approached by a member of the research team. Once written consent had been provided, interviews were undertaken. The first 11 interviews, specifically focusing on individuals with CMH needs, took place between September 2021 and May 2022; these were undertaken remotely due to COVID-19 restrictions. The remaining 14 interviews took place with all participants under the care of mental health services between February 2023 and February 2024. These were in-person interviews conducted at CWP or third sector premises; the interviews took place at two different time points because they were two separate projects, but we chose to combine the findings of the interviews because crisis services were a focus of both.

### Data analysis

With the participant’s permission, discussions were audio recorded, transcribed verbatim, checked against the audio files for accuracy by the researchers who conducted the interviews (L.S. and A.B.) and analysed using grounded theory.^22^ The study adopted an ontologically critical realist approach and was epistemologically objectivist. Participant narratives were accepted as lived realities, rather than actualities, even if their accounts simply represented an interpretation of the events they had experienced. The research team adopted a critical reflexive judgement, accepting that behaviours and perceptions are affected by societal norms and expectations. Because some of the authors have personally experienced a mental health episode, some have provided care for loved ones in crisis and others have no experience of either, we embodied a mix of subjective and objective positions. Transcripts were analysed by the research team, each with different disciplinary backgrounds (P.S., R.N., L.S., A.B., J.T., J.M. and P.A.-M.). The analysis was both iterative and inductive in nature, with rigorous and accurate interpretation of the data maintained through regular meetings between the main analyst and the research team. To establish procedural reliability and conceptual credibility,^24^ additional members of the research team with experience in qualitative methods examined a sample of transcripts to compare their perceptions of the data with the main analyst’s interpretation. Transcripts were hand-coded and subjected to verbatim (line-by-line) coding, followed by descriptive (focused) coding. Following this, analytic codes (super-categories) were derived from the merging of descriptive codes and, finally, interpretative codes (themes) were generated from the collapsing, splitting or reorganisation of analytic codes. The final theory emerged from the interpretation of the relationship between themes. Analysis followed a participant-by-participant format, where the first transcript was analysed fully before the second, and so on.

## Results

There were equal numbers of male (*n* = 12) and female (*n* = 12) participants, and one non-binary participant. Participants were mostly White British (*n* = 24), with a mean age of 39 years. At the time of interview, 7 participants were in-patients and 18 were living in the community. Borderline personality disorder (BPD) was the most common primary diagnosis (*n* = 10), followed by schizophrenia (*n* = 7) and depression (*n* = 3). The interviews lasted between 15 and 79 min, with an average duration of 38 min (see Table 3). Three themes were derived from the grounded theory analysis, with each comprising several super-categories (see Table 4). All participant quotes are accompanied by a pseudonym to ensure anonymity.

**Table 3:** Participant demographics

Pseudonym	Gender	Age (years)	Ethnicity	Primary diagnosis	Placement	Interview duration (min)
Chloe	Female	38	White British	BPD	Community	79
Sarah	Female	22	White British	BPD	In-patient	33
Sam	Male	52	White British	BPD	In-patient	66
Eric	Male	37	White British	Schizophrenia	In-patient	15
Jacob	Male	21	White British	BPD	Community	65
Micah	Male	51	White British	Schizophrenia	In-patient	31
Chelsea	Female	35	White British	Bipolar	In-patient	20
Matthew	Male	37	White British	Schizophrenia	Community	50
Catherine	Female	49	White British	Schizophrenia	Community	21
Trevor	Male	66	White British	Schizophrenia	In-patient	32
Adam	Male	33	White Asian	Schizophrenia	In-patient	45
Athena	Female	33	White British	BPD	Community	43
Linda	Female	43	White British	BPD	Community	42
Roman	Male	39	White British	BPD	Community	36
Jasmine	Female	26	White British	BPD	Community	26
Macauley	Male	39	White British	Schizophrenia	Community	16
Alex	Non-binary	53	White British	ASD	Community	43
Arthur	Male	45	White British	PTSD	Community	37
Paolo	Male	46	White British	Depression	Community	31
Camilla	Female	27	White British	BPD	Community	45
Tina	Female	47	White British	Depression	Community	24
Eliza	Female	57	White British	BPD	Community	44
Joy	Female	53	White British	PTSD	Community	35
Willow	Female	50	White British	Depression	Community	31
Stuart	Male	46	White British	PTSD	Community	39

BPD, borderline personality disorder; ASD, autism spectrum disorder; PTSD, post-traumatic stress disorder.

**Table 4:** Themes and their corresponding super-categories

Grounded theory analysis	
Themes (higher-order themes)	Super-categories (lower-order themes)
More than a diagnosis	Human being, not a diagnosis Stigmatising staff attitudes Viewed as a threat
Instilling hope for the future	Tangible improvements in mental health Sense of community Benefits of creative recovery Saved a life
A safe space for recovery	A safe place for bad days Building trust with staff Therapeutic alliance

### Theme 1: more than a diagnosis

Some participants felt that receiving a mental health diagnosis had been beneficial to their recovery; however, it was important that staff understood that they were more than a label (human being, not a diagnosis). Participants felt that NHS staff often held negative views about people with certain conditions (stigmatising staff attitudes) and that they were treated with caution and even disrespect in environments such as emergency departments (viewed as a threat) because of their diagnosis, while third sector staff were less discriminatory.

### Human being, not a diagnosis

Ten participants explained the importance of having their individual needs and attributes recognised by staff, rather than being viewed as a diagnosis. They discussed how it made them feel ‘valued’ when staff implemented person-centred care and recognised the ‘person as a whole’ rather than ‘making assumptions’ based on their primary diagnosis. Two participants highlighted the importance of their diagnosis in relation to their recovery:

‘I was like, “Okay, this explains so much of my experiences for the past few years,” especially the mood changes, the dissociation, the constant struggle with self-harm, suicidal thoughts. […] I just see it as a gateway to accessing the right therapy.’ – Camilla

However, they wanted staff to recognise that they were ‘more than that’ and disregard preconceptions:

‘You’re not just a diagnosis, you’re not just a history, you’re not just these things, or these medications.’ – Roman

Participants described experiencing a different atmosphere in third sector spaces, where they felt accepted for who they were:

‘You’re not treated like a mental patient when you come here. You’re treated like a human being. You’ve got problems, but hey. You’re amongst friends.’ – Eliza

### Stigmatising staff attitudes

Six participants recalled discrimination by staff due to their diagnosis of BPD, autism or schizophrenia:

‘Some of my diagnoses, there’s a lot of stigma towards them and it’s a stigma that’s held from all levels of professionals. It’s held from support workers, nurses, occupational therapists, psychiatrists, psychologists.’ – Jacob

This was common in emergency departments, where participants felt they were branded as ‘attention-seeking’ and ‘hard to work with’:

‘I’ve been to A&E, and I think it’s more conscious in the back of my mind then that they’re going to see this diagnosis and be like, “It’s just for attention.’” – Camilla

Participants felt they were not taken seriously, even when they had attempted to end their lives. They believed staff viewed them as a hindrance and secondary to those attending with solely physical ailments. Participants also reported stigma from other NHS staff, such as general practitioners and care coordinators, which they felt resulted from a ‘lack of awareness’ about diagnoses such as BPD and autism:

‘It would be like, “Why have you been in A&E? You shouldn’t use these services like that. You shouldn’t be taking overdoses.” And I was like, “I know I shouldn’t be doing it, but I’m not very well.” It was the only way I could communicate.’ – Athena

### Viewed as a threat

Participants described feeling judged by staff when attending emergency departments in a mental health crisis. They felt that staff were guarded towards them, particularly when they had self-harmed, which contrasted with their experiences of attending crisis cafés, where they felt accepted due to the ‘homely’ environment:

‘It’s not as personable, A&E. […] You’re sort of more seen as a threat.’ – Roman

This was concerning for participants, who were simply seeking a safe place to be cared for during a time of psychological distress.

### Theme 2: instilling hope for the future

Participants described how feeling hopeful was important for recovery. Despite previously feeling as though suicide was their only option, certain services had instilled them with hope that their situation would improve, resulting in positive treatment outcomes such as improved relationships, less reliance on psychotropic medication and reduced isolation (tangible improvements in mental health, sense of community). Crisis cafés were particularly beneficial, because participants could engage with non-clinical treatment to increase their skills (benefits of creative recovery) and remind themselves of their capabilities (saved a life).

### Tangible improvements in mental health

Of the five participants who reported substantial improvements to their well-being, four credited the changes to crisis cafés while one was thankful for acute in-patient care:

‘I thought I was beyond help, but I’ve managed to get the help I need. Psychologically and emotionally, now, I’m resilient. I can problem solve. Before I came to hospital, I was very fragile.’ – Sam

When discussing experiences of engaging with third sector support, including crisis cafés and well-being recovery projects, participants reported learning coping strategies, feeling less isolated and breaking negative behaviour patterns:

‘I’ve tried absolutely everything. And, so far, these wonderful people are the only thing that’s working. I mean, okay, I’m still taking medication, but it’s been reduced for the first time in over 20 years.’ – Eliza

### Benefits of creative recovery

Something unique offered by crisis cafés were creative recovery sessions with which people could engage as a form of ‘postvention’ once they were no longer a danger to themselves. According to participants, this could be anything from group journalling to candle painting, where the aim was to empower recovery through creative arts, holistic therapies or physical movement. Three participants were involved not only as members, but also as volunteers:

‘When you see somebody’s face change to a different expression after they’ve finished the group, it’s priceless. It means they’ve done something and enjoyed it and that’s what it’s about, enjoying things again in a creative way.’ – Alex

One participant reported that journalling had led to her becoming ‘mentally well’, while another explained how learning to play the cornet reduced his physical symptoms of anxiety:

‘Because it’s playful, your brain, little by little, does it, and it helps a lot with memory, brain fog, again nervous systems, to cope with that, get away from this sympathetic mode to a less “fight” state.’ – Arthur

While one participant acknowledged that creative recovery alone could not ‘dig into the trauma of somebody from 30 years ago’, participants explained how engaging with the sessions had increased confidence in their skills and instilled hope for the future. One participant enjoyed engaging with music lessons to the extent that he was considering training to become a guitar teacher, which gave him a goal to work towards.

### Sense of community

Participants described a sense of belonging in third sector services that they did not feel existed in NHS environments. They were able to connect with other charity members at crisis cafés, which reduced loneliness and harboured friendships:

‘Without knowing the inside and out of what people are going through, you get a feeling that you’re going through similar things.’ – Stuart

‘All the women in the group are now helping each other and egging each other on and, “You’re doing well, come on,” and we’ve got our own little chat room.’ – Tina

Those who volunteered at creative recovery sessions explained how supporting others made them feel hopeful and validated their skills:

‘If I can have a conversation and use my experience with mental health to give somebody else just a little bit more positivity… […] You’re not going to get that in a clinical setting. It’s in this more personal, less formal situation.’ – Paolo

### Saved a life

Eight participants reported that engaging with the right mental health support had saved their lives. For six of these, the support was provided by the third sector after years of unsuccessful engagement with NHS services:

‘It has literally saved my life. I would have killed myself had it not been for this service. […] That is something you get from a place like this, that there is a way out.’ – Paolo

‘I’ve got my life back. There are still hiccups along the way, but those hiccups don’t involve me trying to end myself. […] I’m feeling more like me than I have in a very long time.’ – Eliza

Some participants credited NHS staff, such as care coordinators or mental health nurses, with saving their lives. Common aspects of these therapeutic relationships were mutual respect, a proactive attitude and genuine empathy:

‘If it wasn’t for them, I wouldn’t be here. It’s the ones that genuinely care and take time to talk to you, to get to know you, to help you with things that you’re struggling with. You don’t ever forget those staff members.’ – Chloe

### Theme 3: a safe space for recovery

Participants described difficulties they had faced in their lives, with adverse childhood experiences a common feature of our sample. Almost half of the cohort reported more than one adverse childhood experience, such as abuse or neglect, and 64% reported a suicidal history.^24^ When they found a service that accepted them (a safe place for bad days), they treasured it and attended frequently, particularly for out-of-hours support. The ongoing care and respect provided by third sector staff supported patients in feeling safe and contained (continuity of care, therapeutic alliance).

### A safe place for bad days

Although some participants reported positive experiences of engaging with NHS services, such as talking therapies and in-patient care, these spaces were criticised for their sterile and clinical atmosphere. Emergency departments were described as unsuitable for accommodating mental health crises:

‘At [hospital name], there are three rooms that are generally used for people who are struggling with mental health, […] but it’s still within A&E, so you can still hear the alarms. You can still hear people in pain.’ – Camilla

This contrasted with the ‘non-clinical’ and ‘easily accessible’ environment of crisis cafés. Participants enjoyed the fact that they could attend at any time without an appointment, even late at night. Participants described crisis cafés as their ‘safe space’:

‘I’ve definitely accessed support here when I’m at my absolute breaking point. […] You can ring up and somebody will be there, no matter what.’ – Camilla

Two participants explained how attending crisis cafés had reduced their self-harm, because they provided a safe outlet and distraction and reduced the potential burden on loved ones:

‘I struggle with self-harm. […] Going somewhere to talk about the fact that I’m having these thoughts takes me out of the situation, takes me out of the home environment where I’ve got the opportunity to do it.’ – Camilla

### Continuity of care

Four participants reported finding it difficult to trust people due to traumatic past experiences, which meant that building a therapeutic alliance with staff took time. They explained how difficult it was to ‘drop [their] guard’, meaning that it was distressing when they had established a rapport with a staff member only to be faced with a different person at their next appointment:

‘The first person that visited me at home, I really clicked with, and he visited me the first couple of times, and then it was different people all the time. I just constantly felt like I was starting again.’ – Paolo

Participants reported being ‘passed from pillar to post’ when engaging with NHS support, mostly due to extensive waiting lists and a lack of specialised support for complex diagnoses. They discussed how it was common to be informed that they were ‘too complex’ for talking therapies and that they would have to wait years to access suitable treatment. They felt there was a lack of support provided by the NHS during the period when they were waiting to access therapy, which left them feeling isolated. Luckily, crisis cafés were able to manage their needs in the meantime, offering an open-door policy, ongoing sessions and a familiar workforce, although they were unable to provide psychological treatment. Crisis lines were also reported as beneficial.

### Therapeutic alliance

The importance of strong therapeutic relationships with staff was highlighted as imperative for recovery. Key aspects of positive relationships were mutual respect, empathy and a non-judgmental attitude:

‘He’s a very good psychiatrist. He does a lot of positive risk taking. […] He was very much on board with me doing as much as I possibly can.’ – Jacob

Participants found it beneficial when staff were ‘on their level’ – for example, when NHS staff were on a ‘first name basis’ with them. It was important for patients to exercise autonomy about treatment decisions via co-production with staff:

‘As long as both parties listen, it should work well. […] We’re not doctors, but we can add a little bit into the discussion and hopefully arrive at something that works.’ – Linda

An aspect of third sector support that participants enjoyed was the willingness of staff to discuss their own lived experience of mental health issues. They did not experience this when engaging with the NHS, and it was something they valued:

‘It helps that the staff all have their own experiences, whether they’ve lost somebody to suicide or they’ve been through suicidal thoughts themselves. I think that having that deeper level is really important. […] It’s sometimes easier to form a relationship.’ – Camilla

Participants felt that this sharing of information was validating and normalised their experience. They also valued the amount of time they were given by third sector staff, who never ‘rushed them out after 10 minutes’ and ‘took their time to really listen’.

## Discussion

### Summary of findings

The experiences of individuals utilising mental health services in the UK can offer useful insight into the efficacy of current provision and assist in service evaluation. This study sought to explore the perspectives of people with lived experience of accessing both NHS-based and third sector support, as well as elucidating their overall experiences of care. The inclusion of individuals with CMH needs is important, because they had extensive experience of engaging with a variety of in-patient and community-based crisis support. Overall findings demonstrated that simple additions to mental healthcare, including mutual respect between patients and staff, gaining hope for the future and having access to safe spaces to use during self-defined crises, can have a considerable impact on patient recovery and overall well-being. Although participants reported that these elements were provided by third sector services, these were mostly lacking in NHS environments such as emergency departments, therapy rooms and in-patient wards. For some, engaging with crisis cafés had contributed to positive treatment outcomes such as reduced psychotropic medication dosage, stronger familial relationships and improved confidence. Several participants explicitly stated that engaging with crisis cafés had saved their lives, following years of unsuccessful engagement with NHS support. The main challenges reported were the lack of continuity of care and co-production provided by the NHS, mainly due to high staff turnover and limited sessions.

### Comparisons with the wider literature

Participants reported experiencing structural stigma when accessing mental health services, particularly when attending emergency departments after self-harming. Emergency department staff have been known to use negative terms when discussing individuals who had attended in suicidal crisis, such as ‘attention-seeker’ and ‘cry for help’,^25^ which our participants also reported. A scoping review by Klein et al^26^ found that the care provided by emergency services is often inadequate for meeting the needs of people with complex diagnoses, and that chronic suicidality among those with conditions such as BPD and schizophrenia could signal that they are not receiving effective treatment to assist their recovery, despite ongoing attempts to seek help. Research suggests that people tend to distance themselves from stigmatised populations, and there is evidence to suggest that some clinicians may emotionally distance themselves from individuals with BPD^25^ due to the mental healthcare system’s discourse of ‘untreatability’,^27^ despite compelling literature suggesting that it is a treatable illness that can be managed with the right support.^28^

Participants reported finding it distressing when they were treated solely based on their diagnosis, which previous research has found is likely to remove autonomy and disregard individual needs and experiences.^14^ Participants with complex diagnoses such as BPD, schizophrenia and autism reported experiencing stigma from a range of mental health professionals, with negative attitudes of higher, older management structures likely to have shaped the attitudes of junior staff.^14,25^ BPD and schizophrenia are serious conditions involving problems in the regulation of emotions and suicidality, and their trajectory has been linked to factors such as childhood abuse and insecure attachment,^26^ both of which were prevalent in our sample. As argued by Rivera-Segarra et al,^29^ stigma arises because people with these conditions ‘are simultaneously viewed as if they are both out of control and as if they can control their behaviour’, meaning they are deemed as being attention-seeking and manipulative regardless of how they act when seeking help. This is something our participants also reported, contributing to feelings of marginalisation.

Participants highlighted the importance of person-centred care, continuity of care and strong therapeutic relationships. They particularly valued mutual respect, empathy and a proactive attitude among staff. Small gestures, such as a staff member remembering their birthday, were recalled with great fondness. Similar findings have been reported for positive aspects of mental health placements,^30,31^ with simple gestures of kindness such as an unplanned call or a hand to hold, which may be regarded as trivial in a professional context, being found to make a real difference to someone in crisis.^32^ Participants reported believing that third sector staff were more likely to engage in these behaviours than NHS staff, with research suggesting that they are more prone to burnout due to time restraints, high staff turnover and organisational pressures.^13,14^ The importance of continuity of care in mental health settings has been recognised in the literature, with ongoing personal relationships with staff found to increase feelings of safety, while setbacks and breaks in relationships have been found to contribute to anxiety.^33^ Our participants highlighted how extensive waiting times contributed to worsening symptoms, with other qualitative studies highlighting the importance of timely, consistent and continued support for individuals with CMH needs.^14,34^

A key finding of this study is the importance of creative recovery sessions for building confidence and regaining hope. While participants acknowledged that creative activities alone would not address deep-rooted trauma, they appreciated the opportunity to engage with creative activities in a group setting, with peer mentor support valued highly. Participation in creative activities has been linked worldwide with positive outcomes for people with mental health issues, with one qualitative study finding that creative workshops had long-lasting benefits for participants with respect to improved confidence and increased understanding of symptoms,^35^ while a systematic review found an indication of positive impacts on connectedness, empowerment and identity.^36^ In line with recovery theories, mental health support for people diagnosed with CMH needs should focus more on well-being and social connectivity than clinical symptoms, because creative activities may cause a transformation of the image of dysfunction associated with mental illness while also promoting hope,^37^ which has been recognised as one of the main processes of, and catalysts for, mental health recovery, both in our research and beyond.^38^

**Fig. 1:**
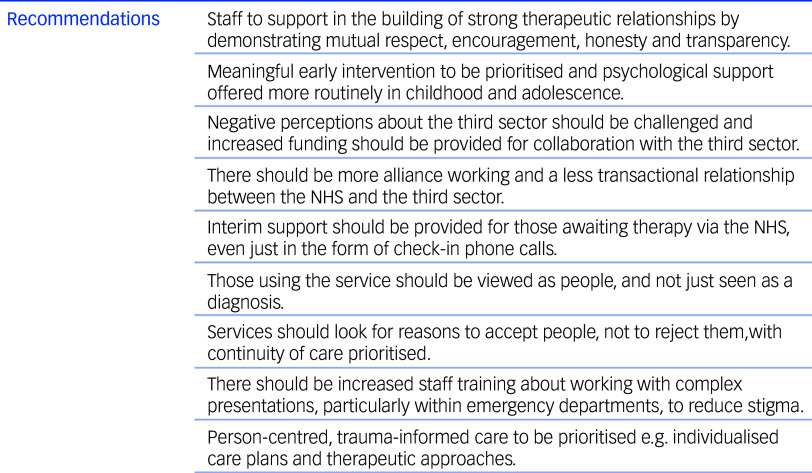
Recommendations for implementation, based on participant interviews. NHS, National Health Service.

This study examines the experiences and perspectives of people accessing crisis support in the UK, and puts forward their recommendations for future practice (see Fig. 1). Although our findings suggest that many individuals were satisfied with the level of care they were receiving for their mental health, it is evident that NHS services are struggling to meet the needs of the population in a timely manner, resulting in lengthy waiting times for therapy and an over-reliance on the third sector. Although there is an expectation that crisis cafés will manage the needs of people while they await NHS treatment, many of these individuals present with CMH needs and require trauma-informed care, which cannot be offered by voluntary staff who are not suitably trained. The NHS Long-Term Plan^6^ identified that crisis cafés are provided at a relatively low cost and result in high satisfaction, which was a sentiment echoed by our participants; however, policymakers must ensure that the NHS is working together with the voluntary sector on these alternatives, and that they will receive adequate funding and do not become overburdened.

### Strengths and limitations

This study is the first qualitative exploration of people’s experiences of third sector crisis support, featuring a comparison between such services and other community-based and in-patient care. A strength of the research is the collection of in-depth data from individuals currently being supported by a range of mental health services in the UK. Access to people using the service provides a closer view of reality, with the findings reflecting ‘real-world’ provision and the experiences of users of these services. The findings should be interpreted in the context of some methodological limitations: for example, interviews were conducted at CWP or third sector premises, which may have introduced selection bias or even social desirability bias, because the location of the interviews may have influenced the participants’ responses. Future research may benefit from being conducted in more ‘neutral’ spaces, such as hired university rooms. Another limitation is that our results may not be representative of the rest of the UK (because data were collected in the north-west of England), although many issues identified are likely to apply across the country. A further limitation is the lack of diversity in our sample, because all but one of the participants were White British, with limited participation from ethnic minority communities. It is important to note that our numbers are largely representative of the ethnic background of the local community, with under 5% of Cheshire and Wirral residents classified as being from ethnic minority groups.^39^ Despite this, we should aim to represent the wider population of the UK in our research, of whom 14% are ethnic minorities,^40^ and ensure that all views are captured moving forward. Specific targeting of certain ethnic groups will improve future research and work to reduce mental healthcare disparities.^40^

## Supporting information

Sambrook et al. supplementary material Sambrook et al. supplementary material

## Data Availability

The data that support the findings of this study are available from the corresponding author, L.S., upon reasonable request.

## Supplementary material

The supplementary material can be found at https://doi.org/10.1192/bjo.2025.30
