# Supplementary material for: ‘I would have killed myself had it not been for this service’: qualitative experiences of NHS and third sector crisis care in the UK
Source: BJPsych Open. 2025 May 14;11(3):e107. doi: 10.1192/bjo.2025.30 (PMC12089819; doi:10.1192/bjo.2025.30)
Supplement: Sambrook et al. supplementary material [file S2056472425000304sup001.docx]

**Appendix**

**Appendix 1. Interview schedule for service users.**

You are being invited to take part in this project which is being conducted by Liverpool John Moores University and Cheshire and Wirral Partnership NHS Foundation Trust. The project aims to explore service users’ experiences of the new models of care adapted within the transformation programme via 1:1 interviews.

Before you decide, it is important for you to understand why the project is being carried out and what it will involve. **Please take time to read the following information carefully and discuss it with others if you wish.** Ask us if there is anything that is not clear or if you would like more information. Take time to decide whether or not you wish to take part.

**Interview questions:**

1. Can you tell me about your experience of attending hospital in a crisis?
2. Can you tell me about your experience of contact with mental health services more generally?
3. What are your thoughts about the care you received for your mental health condition?
4. If you have been an inpatient, how did you find the decisions that were made in respect of your care?
   1. What was your involvement in the decision-making process?
   2. Did you experience a sense of autonomy in respect of your care? Why/why not?
   3. Could you tell me more about the psychological therapies you received?
   4. Were you prescribed psychotropic medication? Could you tell me more about your experience of this?
   5. Did you feel prepared to be discharged? Why/why not?
   6. How would you describe your relationships with staff? Did any of these relationships continue afterwards e.g. with social workers? Were these relationships consistent? Why/why not?
   7. Have you ever used the crisis line? If yes, how was your experience?
   8. Have you ever utilised a crisis café? If yes, how was your experience?
5. If you have been cared for by the Community Mental Health Team (CMHT), how was your experience of this?
   1. What was your involvement in the decision-making process?
   2. Did you experience a sense of autonomy in respect of your care? Why/why not?
   3. Could you tell me more about the psychological therapies you received?
   4. Were you prescribed psychotropic medication? Could you tell me more about your experience of this?
   5. How would you describe your relationships with staff? Did any of these relationships continue afterwards e.g. with social workers? Were these relationships consistent? Why/why not?
6. Overall, in terms of your experience of contact with mental health services and the care you received, what helped and what was not as helpful?
7. In terms of positive experiences, why do you think these aspects were successful?
8. In terms of negative experiences, why do you think these aspects were unsuccessful? Were there placements that did not work? If so, why?
9. Do you have any suggestions about possible improvements that could be made?
10. Is there anything else you would like to discuss?
